# Hybrid Agent-Based Modeling of *Aspergillus fumigatus* Infection to Quantitatively Investigate the Role of Pores of Kohn in Human Alveoli

**DOI:** 10.3389/fmicb.2020.01951

**Published:** 2020-08-12

**Authors:** Marco Blickensdorf, Sandra Timme, Marc Thilo Figge

**Affiliations:** ^1^Research Group Applied Systems Biology, Leibniz Institute for Natural Product Research and Infection Biology – Hans Knöll Institute, Jena, Germany; ^2^Faculty of Biological Sciences, Institute of Microbiology, Friedrich Schiller University Jena, Jena, Germany

**Keywords:** virtual infection modeling, *Aspergillus fumigatus* lung infection, Pores of Kohn, human model, hybrid agent-based computer simulations

## Abstract

The healthy state of an organism is constantly threatened by external cues. Due to the daily inhalation of hundreds of particles and pathogens, the immune system needs to constantly accomplish the task of pathogen clearance in order to maintain this healthy state. However, infection dynamics are highly influenced by the peculiar anatomy of the human lung. Lung alveoli that are packed in alveolar sacs are interconnected by so called Pores of Kohn. Mainly due to the lack of *in vivo* methods, the role of Pores of Kohn in the mammalian lung is still under debate and partly contradicting hypotheses remain to be investigated. Although it was shown by electron microscopy that Pores of Kohn may serve as passageways for immune cells, their impact on the infection dynamics in the lung is still unknown under *in vivo* conditions. In the present study, we apply a hybrid agent-based infection model to quantitatively compare three different scenarios and discuss the importance of Pores of Kohn during infections of *Aspergillus fumigatus*. *A. fumigatus* is an airborne opportunistic fungus with rising incidences causing severe infections in immunocompromised patients that are associated with high mortality rates. Our hybrid agent-based model incorporates immune cell dynamics of alveolar macrophages – the resident phagocytes in the lung – as well as molecular dynamics of diffusing chemokines that attract alveolar macrophages to the site of infection. Consequently, this model allows a quantitative comparison of three different scenarios and to study the importance of Pores of Kohn. This enables us to demonstrate how passaging of alveolar macrophages and chemokine diffusion affect *A. fumigatus* infection dynamics. We show that Pores of Kohn alter important infection clearance mechanisms, such as the spatial distribution of macrophages and the effect of chemokine signaling. However, despite these differences, a lack of passageways for alveolar macrophages does impede infection clearance only to a minor extend. Furthermore, we quantify the importance of recruited macrophages in comparison to resident macrophages.

## Introduction

External cues constantly threaten the healthy state of organisms. Due to the daily inhalation of hundreds of particles and pathogens the immune system needs to constantly accomplish the task of pathogen clearance in the lung in order to maintain and restore a healthy state. However, infection dynamics are highly influenced by the peculiar anatomy of the human lung. In 1893, Kohn described inter-alveolar pores in a pneumonia patient for the first time (Kohn, 1893). In the following years, Oertel was the first to point out that these pores, nowadays known as Pores of Kohn (PoK), may open and close due to pressure changes during respiration (Oertel, 1919). Although PoK were considered to possibly contribute to infection processes (Adams and Livingstone, 1931), the scientific community lacked evidence for this until Cordingley (1972) characterized PoK using electron microscopy. In this work, alveolar macrophages (AM) – i.e., resident phagocytes in the lung – were observed inside a PoK supporting the postulated role of PoK as inter-alveolar cellular communication channels. Furthermore, a low number of PoK, as occurs in infants, increases the risk of atelectasis, whereas an increase in the number or size of PoK, as seen in older individuals or as caused by smoking, is associated with a higher risk of emphysema (Wright, 2001; Rennard et al., 2006; Guillerman, 2010; Yoshikawa et al., 2016). Today, the important role of PoK for ventilation is widely accepted (Namati et al., 2008), although the exact function of PoK in the lung is still not fully understood and experimental evidence supports partially contradicting hypotheses. If PoK are regulators of air pressure and thus open and close at a typical human respiration rate of approximately 3–5 s (Barrett et al., 2012), how can they serve as entry/exit points for immune cells, such as AM, which migrate at a much lower pace? How do PoK allow for collateral ventilation when covered with surfactant (Oldham and Moss, 1989)? In case AM are indeed routinely migrating between neighboring alveoli through PoK, how does this affect the dynamics of infection processes?

In this study, we want to investigate the function of PoK during *Aspergillus fumigatus* infection in the human lung. *A. fumigatus* is a human pathogenic mold, which can cause severe infections in immunocompromised patients (Latgé, 1999, 2001; Brakhage et al., 2010). The small conidia of *A. fumigatus*, which are 2–3 μm in size, are able to overcome the various filter mechanisms, such as the cilia and the mucous layer (Bustamante-Marin and Ostrowski, 2017), of the respiratory tract and most probable reach the alveoli, which constitute the majority of the lung surface (Weibel, 1963; Latgé, 1999; Brakhage et al., 2010). Once the conidia are embedded in the surfactant, which covers the alveolar epithelial cells (AEC), they begin to swell and a complex host-pathogen response is initiated (Behnsen et al., 2008; Margalit and Kavanagh, 2015). It can be assumed that shortly after arrival of a conidium in the alveolus, the complement system within the surfactant layer will be activated. This will be sensed by the AEC on which the conidium is located and will lead to their secretion of chemokines. Thus, complement activation and AEC chemokine secretion provide a first line of defense recruiting AM for conidia uptake (Pollmächer and Figge, 2014; Margalit and Kavanagh, 2015). If not cleared within the first 6 h (Baltussen et al., 2018), *A. fumigatus* conidia develop hyphae, which in turn may invade the bloodstream, causing dissemination and severe infections (Van De Veerdonk et al., 2017). Therefore, *A. fumigatus* represents an interesting target to study the role of PoK during infection. Although methods for studying pulmonary tissue, such as electron microscopy, confocal laser endomicroscopy (Danilevskaya et al., 2015), *ex vivo* techniques (Hocke et al., 2017) or the emerging lung-on-chip models (Mosig, 2017) do exist, it is not yet possible to capture the full cellular and molecular dynamics during infection in alveoli in living organisms. A systems biology approach allows to address questions that cannot be answered by traditional wet-lab experiments (Horn et al., 2012; Medyukhina et al., 2015; Deinhardt-Emmer et al., 2020; Schicke et al., 2020). Especially for complex infection processes, where cellular and molecular as well as temporal and spatial dynamics may be of importance for in-depth understanding of host-pathogen interactions, virtual modeling provides a valuable tool. This has been demonstrated by established models based on ordinary differential equations (ODE) (Hancioglu et al., 2007; Voit, 2014) or partial differential equations (PDE) (Hao et al., 2015; Sharp et al., 2015) as well as state based models (Lehnert et al., 2015; Prauße et al., 2018; Sreekantapuram et al., 2020). In processes where resolution of individual cells is of importance, as in the case of *A. fumigatus* infection, agent-based models are most promising to fully capture the spatio-temporal infection dynamics (Tokarski et al., 2012; Lehnert et al., 2015; Timme et al., 2018). Therefore, we here applied a previously developed hybrid agent-based virtual infection model (Pollmächer and Figge, 2014, 2015; Blickensdorf et al., 2019), which incorporates the immune cell dynamics of alveolar macrophages as well as the molecular dynamics of diffusing chemokines that are secreted by AEC and attract alveolar macrophages to the site of infection. In this way, it enables the comparison and quantification of possible functions of PoK. Therefore, we setup three different model scenarios where PoK serve as passage points for (i) AM and chemokines referring to the hypothesis of AM passaging through PoK from Oertel (1919) and Cordingley (1972) (ii) chemokines but not AM and (iii) neither AM nor chemokines as postulated by Namati et al. (2008).

## Materials and Methods

### Existing Modeling Framework

**FIGURE 1:**
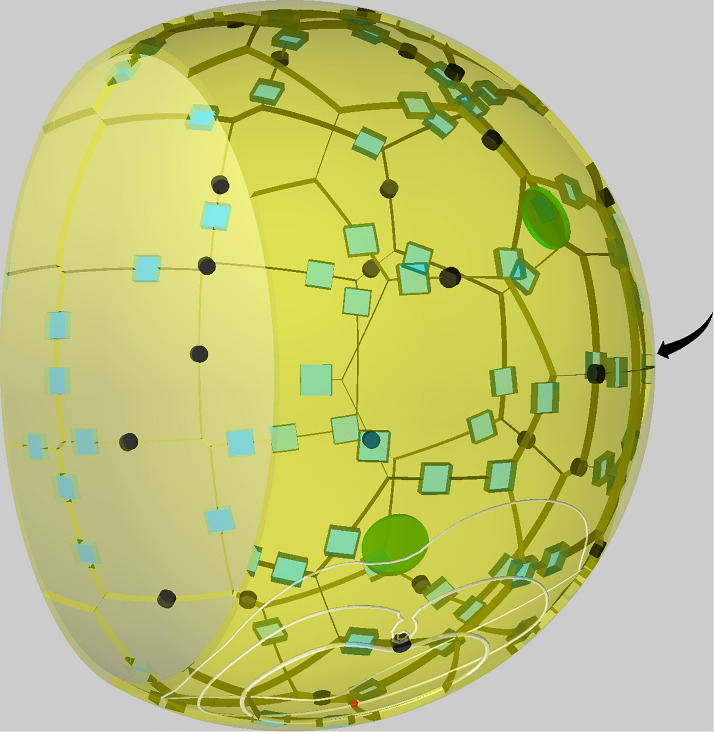
Visualization of a to-scale human alveolus in the hybrid agent-based model. The alveolar entrance ring (left) and Pores of Kohn (black) represent entry/exit points for alveolar macrophages (green) and chemokine flow (white isolines) induced by the alveolar epithelial cell where the conidium (red) is located. Alveolar surface is covered with epithelial cells of type 1 (yellow) and type 2 (blue). The pole of the alveolus is indicated by the arrow.

In order to investigate the role of PoK in human alveoli and, in particular, their impact on the migration and detection dynamics of AM during *A. fumigatus* infections, we performed simulations with a previously developed hybrid agent-based modeling framework (Pollmächer and Figge, 2014, 2015). This model approximates the shape of an alveolus by a sphere (see Figure 1) that is cut at 3/4 of its diameter forming an alveolar entrance ring, which represents its connection within an alveolar sac of the human lung. The alveolus is composed of AEC of type 1, which are placed equidistant over the surface using a Voronoi tessellation, and of type 2, placed along the edges of type 1 AEC. PoK that represent connections between neighboring alveoli are positioned in the same way. Parameters of e.g. cell size and numbers were obtained from an in-depth literature search and can be found in Table 1.

**TABLE 1:** Default model parameters.

**Parameter**	**Description**	**Value**
*n*_*AM*_	Number of AM per human lung	2.1×10^9^ (Wallace et al., 1992)
*n*_*Alv*_	Number of alveoli per human lung	4.8×10^8^ (Ochs et al., 2004)
*r*_*AM*_	Radius of AM	10.6μm (Krombach et al., 1997)
*n*_*AEC1*_	Number of alveolar epithelial cells type 1 per alveolus	39–45 (estimation)
*n*_*AEC2*_	Number of alveolar epithelial cells type 2 per alveolus	74–84 (estimation)
*n*_*PoK*_	Number of pores of Kohn per alveolus	24 (Kawakami and Takizawa, 1987) (estimation)
*r*_*PoK*_	Radius of pores of Kohn	2.00μm (Kawakami and Takizawa, 1987)
*r*_*Alveolus*_	Radius of an alveolus	116.5μm (Balásházy et al., 2008)

Our previous studies used an agent-based framework showing that a passive movement of conidia by respiration does not delimit infection clearance, whereas a higher speed and/or persistence time of AM improves detection of *A. fumigatus* conidia. However, for reasonable parameter ranges the infection was not cleared in more than 20% of the simulations (Pollmächer and Figge, 2014). Therefore the model was extended by chemokine secretion in a hybrid agent-based model. An investigation of the chemokine parameters revealed, that a high ratio of secretion rate to diffusion coefficient facilitates AM to detect the conidium much faster (Pollmächer and Figge, 2015).

In the current study we applied this model to infections of *A. fumigatus*, whose conidia can reach an alveolus and, if not phagocytosed, swell and develop hyphae that can invade the bloodstream leading to severe infections with high mortalities of up to 90% (Dagenais and Keller, 2009). To simulate this infection scenario, we placed a single conidium into the virtual alveolus at a random position. This is justified by the large number of alveoli per human lung, making the case of one conidium per alveolus the most probable infection scenario (Pollmächer and Figge, 2014). The number of AM inside one alveolus is binomially distributed according to the number of alveoli *n*_*Alv*_ and the number of AM in the human lung *n*_*AM*_ and yield a mean number of 4.38 AM (Wallace et al., 1992). We infer from this distribution $B_{AM}=\left(n_{AM},p,k\right)=\left(\begin{array}{c} n_{AM}\\ k \end{array}\right)p^{k}{\left(1-p\right)}^{n_{AM}-k}$ the initial number of AM inside the alveolus at simulation start with $p=\frac{1}{n_{A\,l\,v}}$. AM perform a biased persistent random walk with persistence time of *t*_*P*_ = 1 min and a speed of *v* = 4μm/min. AM may leave the system if they, by chance, cross the border of the alveolus at either the alveolar entrance ring or at a PoK. To maintain a constant average number of AM in the alveolus, new AM are inserted at the model boundary after an exponentially distributed waiting time of $t=1/\lambda_{i\,n}\,\ln\left(\frac{1}{u}\right)$ with *u* uniformly distributed in [0,1) and input rate λ_*in*_, which has to be calibrated for each AM speed, persistence time and model boundary in the healthy state i.e., without present pathogens.

The hybrid agent-based model also comprises chemokine signaling released by the AEC where the conidium is located. The chemokine diffuses at the inner alveolar surface forming a chemokine gradient that guides the AM toward the chemokine-secreting AEC on which the conidium is located. To simulate chemokine signaling we included a reaction-diffusion model based on PDE in the agent-based framework of the alveolus. The equation $\frac{\delta \,c\,\left(\vec{r},t\right)}{\delta \,t}=D\,\delta \,c\,\left(\vec{r},t\right)+S\,\left(\vec{r},t\right)-Q\,\left(\vec{r},t\right)$ incorporates chemokine secretion by the source term *S* and chemokine diffusion with diffusion coefficient *D* for chemokine concentration $c\,\left(\vec{r},t\right)$ at point $\vec{r}$ and time point *t*. Chemokines are secreted over the whole surface of the pathogen-associated AEC with constant secretion rate *s*_*AEC*_. The chemokine uptake by AM with term *Q* is realized with a spherical adaption of the receptor ligand-model of Guo et al. and Guo and Tay (Riedemann et al., 2002; Guo et al., 2008), in which chemokine may be bound to AM surface receptors. This allows AM to sense the chemokine gradient, during a persistence time *t*_*P*_, to obtain a weighted cumulative gradient. AM change their direction biased with a probability to follow the gradient, which is proportional to the receptor differences along the weighted cumulative gradient after *t*_*p*_. Hence, AM are able to sense the chemokine gradient and adapt their migration behavior accordingly after expiration of the persistence time. The boundaries of this system, i.e., the alveolar entrance ring and the PoK, form sinks allowing for chemokine outflow. Furthermore, the presence of chemokines in the system affects the probability of an AM to enter the alveolus at a certain boundary position. The higher the chemokine concentration at this position is, the higher is the probability that a new AM enters the alveolus at this site. The diffusion equation is solved by an implementation of the finite difference method for unstructured grids (Sukumar, 2003). As grid a near–equidistant Voronoi tessellation was created as a solution of the Thomson Problem (Thomson, 1904) by use of an algorithm implemented by MacWilliam and Cecka (2013).

Finally, in order to evaluate the infection outcome of a simulation we compute the infection score *IS*, which we define as the fraction of 1000 simulations, in which the conidium was not detected by AM within the first 6 h, i.e., the typical time needed for *A. fumigatus* to swell and start developing hyphae (Pollmächer et al., 2016).

### Study Setup to Investigate the Role of PoK

In this study we want to investigate the impact of PoK on the infection dynamics in human alveoli. In order to do so, we setup three types of infection scenarios (see Supplementary Videos 1–3). The first setup represents the situation, in which PoK serve as entrance and exit points for AM and for the flow of chemokines through PoK. This setup corresponds to the standard model used in our earlier studies and we here refer to it as PoK+/+ model. It reflects the hypothesis that immune cells use PoK as migration channels between neighboring alveoli. However, since it is not experimentally verified *in vivo* that AM do indeed migrate through PoK, we setup a second model, which we refer to as PoK+/− model. In this scenario chemokines can still flow through PoK, while AM do not enter or exit through PoK. Furthermore, in the third setup neither AM can migrate through the PoK nor chemokine can flow out at PoK. Thus, we refer to this scenario as PoK−/− model. However, since conidia do not migrate actively and it is rather unlikely that they are located directly on a PoK when entering the alveoli, we do not consider passing of conidia through PoK in our model. In our study, we will quantitatively compare these three scenarios by computer simulations.

The models PoK+/− and PoK−/− have system boundaries that are decreased relative to the PoK+/+ model. This is associated with a decreased probability of AM leaving the alveolus during the course of the infection scenario. Thus, we adapted the respective AM input rate λ_in_ for the three different scenarios. As found previously, timely *A. fumigatus* clearance in the human alveolus is determined mostly by the chemokine parameters (Pollmächer and Figge, 2015). In particular, we showed that the most important chemokine parameters are the secretion rate *s*_*AEC*_ and the diffusion coefficient *D*(Pollmächer and Figge, 2015). Since the physiological values for these parameters could not yet be measured in experiment, we scan for a broad range of physiologically reasonable values in our computer simulations. It is generally observed that the combination of a high value of *s*_*AEC*_ and a low value of *D* is beneficial for the fast conidium detection by AM. In contrast, if the ratio of *s*_*AEC*_/*D* is too low, chemotactic signaling loses its unique function of guidance and AM are unable to find the conidium within 6 h, as is the case for a search by random walk (Pollmächer and Figge, 2014, 2015).

## Results

In the present study we investigated the impact of PoK on the infection dynamics during *A. fumigatus* lung infection. In order to do this, we constructed three different models that account for different properties of PoK, such as entry and exit points for AM as well as chemokine outflow. These three models are (i) the PoK+/+ model, where AM can enter and exit through PoK and chemokines can flow out at PoK, (ii) the PoK+/− model, where AM cannot enter and exit through PoK but chemokines can flow out at PoK, and (iii) the PoK−/− model, where PoK serve neither as migration channels for AM nor as sinks for chemokine outflow (see Supplementary Videos 1–3). However, we assume that, if AM are able to migrate though PoK, also the much smaller chemokine molecules can diffuse through them. Therefore, we do not consider a scenario, where PoK serve as migration channels for AM while chemokine outflow is not possible.

It may be expected that, since the recruitment of AM through the homogeneously distributed PoK everywhere in an alveolus allows for shorter AM migration distances in the PoK+/+ model, this model yields significantly lower infection scores compared to the models PoK+/− and PoK−/−. In the latter models, AM enter the alveolus exclusively via its entrance ring, which may be associated with longer migration distances toward the conidium and thus extended search times. In addition, in the PoK−/− model chemokine diffusion might be affected in two different ways: First, the overall chemokine concentration in the alveolus will be higher compared to the PoK+/+ and PoK+/− model, because chemokines cannot flow out at PoK. As shown previously, a high secretion is beneficial for infection clearance by AM (Pollmächer and Figge, 2015), thus less chemokine outflow and therefore a higher chemokine concentration might also be beneficial for AM searching a conidium. Secondly, PoK provide a sink for the chemokine and thus locally distort the chemokine profile around the PoK, which may guide AM away from PoK following the locally distorted gradient. Once the distance to the PoK has increased, AM get re-directed toward the conidium. Thus, it may be expected that the absence of distortions in the chemokine profile, as in the PoK−/− model where chemokines do not flow out of PoK, could have an additional positive effect on infection clearance.

The Results section is structured as follows: In Section “Reduced Length of Alveolar Boundary Alters Spatial Equilibrium Distribution of AM,” we analyze the calibration of the AM input parameter for each model scenario and the spatial redistribution of AM resulting from the different conditions in the three scenarios. Next, in Section “PoK+/+ Shows Highest Infection Clearance Compared to PoK+/− and PoK−/−” we investigate the infection clearance in the three scenarios for various chemokine parameters and how this depends on the position of the conidium in the alveolus. Moreover, we analyze the contribution of AM recruitment to infection clearance in comparison with resident AM that are present in the alveolus from the start of the simulation. In Section “Presence of PoK Prevents Chemokine Accumulation,” we first compare the optimal chemokine parameters that we identify for each of the three scenarios. Secondly, we study the effect of chemokine accumulation that is observed for the PoK−/− model. Thirdly, we assess the impact of the different chemokine profiles of the three scenarios on the migration pattern of AM. Finally, in Section “Accumulation of AM Limited in Presence of PoK Passageways” we investigate the effect of AM accumulation that is observed during the simulations and is caused by the chemokine secretion by AEC. We analyze this accumulation under comparable conditions for all three model scenarios with the conidium being placed at the pole of the alveolus.

### Reduced Length of Alveolar Boundary Alters Spatial Equilibrium Distribution of AM

**FIGURE 2:**
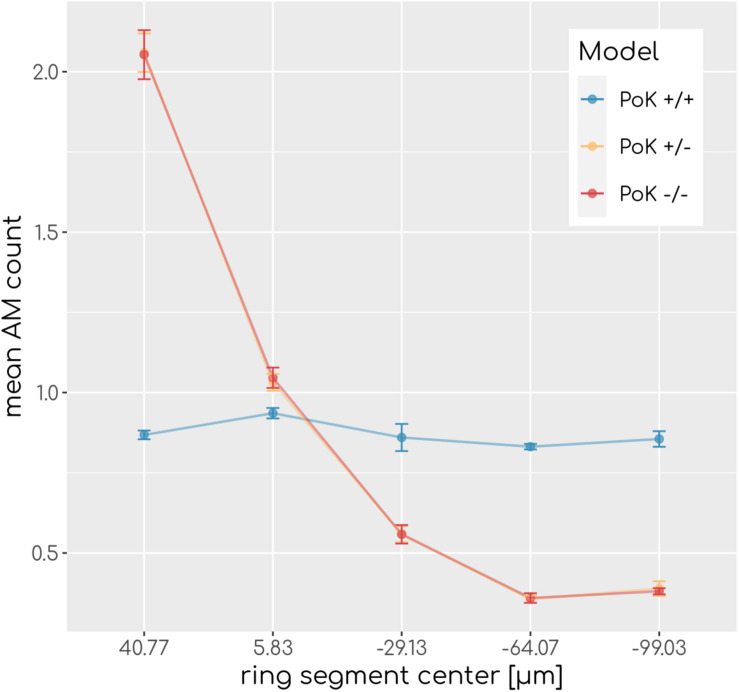
Spatial AM distribution. Mean AM count after equilibration of system dynamics along the main axis of the alveolus from the entrance ring (at 59 μm) to the pole (at -116.5 μm). Each data point refers to an average over ring segments with equal area of 25,583 μm^2^.

In the PoK+/− and PoK−/− model, where AM only enter/exit the alveolus at the alveolar entrance ring but not at PoK, the length of the system boundary is effectively shorter in comparison to the PoK+/+ model, the re-calibration of the AM input rate λ_*in*_in the absence of infection (for details see “Materials and Methods” section) was found to be associated with an altered spatial equilibrium redistribution of AM at the inner surface of the alveolus. To be more specific, the initial random spatial distribution of AM over the alveolar surface in the PoK+/+ model was altered into a spatial equilibrium distribution for the PoK+/− and the PoK−/− model, where AM appear accumulated at the alveolar entrance ring (see Figure 2). Interestingly, due to the absence of any conidia in the human alveolus during the calibration of λ_in_ (see section “Existing Modeling Framework”), this is a direct consequence of excluding PoK as part of the system boundary, where AM can leave and enter the alveolus; thus, turning the alveolus into a spatial dead end for randomly migrating AM in the PoK+/− and the PoK−/− model.

### PoK+/+ Shows Highest Infection Clearance Compared to PoK+/− and PoK−/−

As measure for infection clearance we determine for each simulation the time point of first contact between an AM and the randomly positioned conidium in the alveolus; this defines the so-called first passage time (*FPT*). The infection score *IS* is given by the fraction simulations with *FPT* > 6*h*, i.e., the number of 1000 simulations, where AM were unable to clear the conidium within 6 h after entrance in the alveolus. This time duration corresponds to the typical time frame until germination of the conidium starts and the fungus becomes invasive; i.e., *IS=0* corresponds to infection clearance in all and *IS=1* corresponds to an infection clearance in no simulation within the first 6 h of infection duration. For comparison of the three different infection models we performed simulations of *A. fumigatus* infections by placing one conidium at a random position in the alveolus and by repeating this scenario for various sets of chemokine parameters. In particular, we screened the secretion rate for values *s*_*AEC*_ = {1500,5000,15,000,50,000,150,000,500,000} min^−1^ and the diffusion coefficient over the range of values *D* = {20, 60, 200, 600, 2000, 6000}μm^2^min^−1^. This range of values is motivated by estimations for chemokine diffusion in lung surfactant and in water (see Supplementary Material Section 1.2).

**FIGURE 3:**
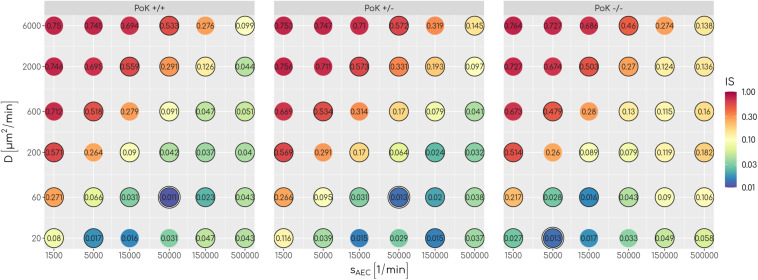
The infection score represented in a color-coded fashion as a function of all scanned combinations of chemokine parameters. Numbers represent the respective infection scores. A black circle around the point indicates significant differences between the three models as tested with a log-rank test.

An analysis of the infection score over all scanned parameters of chemokine signaling revealed that low values with *IS*∼0.01equivalent to clearance of the infection can be achieved for all three models, albeit for different optimal chemokine parameters. For a deeper understanding of the differences between the models, we compared the*FPT* distributions of the three models by a survival analysis using a log-rank test for each combination of chemokine parameters (see Supplementary Material Section 1.1). This analysis revealed that for 17 out of 36 parameter combinations significant differences between the models exist (see Figure 3) showing that infection clearance is affected by the function of PoK. In addition, the absolute value of the relative difference between the respective infection scores are above 0.5 for 28% of all scanned chemokine parameters showing that the function of PoK induces a substantial difference for infection clearance.

**FIGURE 4:**
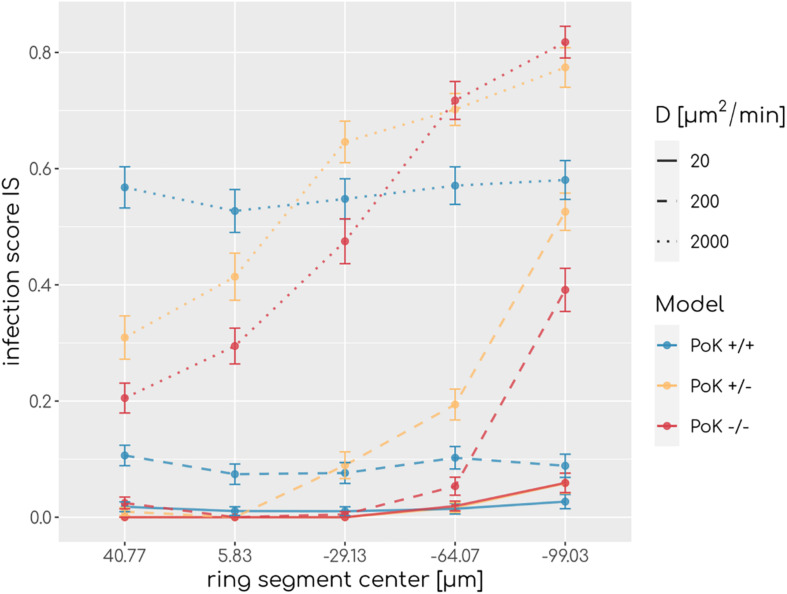
Infection scores along the main axis of the alveolus from the entrance ring (at 59 μm) to the pole (at -116.5 μm) for various diffusion coefficients and secretion rate *s*_*AEC*_ = 15,000*min*^−1^. Each data point refers to an average over ring segments with equal area of 25,583 μm^2^.

To understand how PoK may influence the infection score, we studied in retrospect those AM that successfully detected a conidium (*AM*_*success*_) in relation with their starting point in the alveolus. An AM may either be present in the alveolus from the start of the simulation (*AM*_*resident*_) or may have entered the alveolus during the simulation (*AM*_*recruited*_), either through a PoK or via the alveolar entrance ring depending on the considered PoK model. We calculated the resident ratio of AM, *r*_*resident*_ = *AM*_*resident*_/*AM*_*success*_, yielding that the mean value for *r*_*resident*_ over all simulated chemokine parameters shows only small differences between the models (*r*_*resident*_(*PoK* + / +) = 0.61 ± 0.04,*r*_*resident*_(*PoK* + /−) = 0.58 ± 0.03,*r*_*resident*_(*PoK*−/−) = 0.56 ± 0.06) indicating that the slight majority of conidia are found by AM, which were not recruited to the alveolus but were initially present in the alveolus. In approximately 40% of the infection scenarios the conidium was found by an AM that was recruited during the infection duration of 6 h. In the PoK+/+ model recruitment of (*AM*_*success*_) is realized by 54% through a PoK and by 46% via the alveolar entrance ring (see Supplementary Figures S1–S3). Since recruitment through a PoK is not possible in the PoK+/− and PoK−/− model, the small differences in the infection score compared to the PoK+/+ model suggest, that recruitment through the alveolar entrance ring is adequately replacing the missing passaging though PoK. We expect this effect to be dependent on the precise position of the conidium in the alveolus, i.e., the larger the distance of the conidium from the alveolar entrance ring is, the more beneficial the presence of PoK will be in the PoK+/+ model. To test this hypothesis we split the alveolar surface into five ring segments of equal area from the entrance ring to the pole of the alveolus and computed the IS individually for each of these segments. This analysis revealed that the infection score in the PoK+/− model and the PoK−/− model is smallest when the conidium is located in the proximity of the alveolar entrance ring and increases for locations with higher distances from the entrance ring, whereas for the PoK+/+ model the infection score remains fairly independent of the location of the conidium in the alveolus (see Figure 4).

### Presence of PoK Prevents Chemokine Accumulation

**FIGURE 5:**
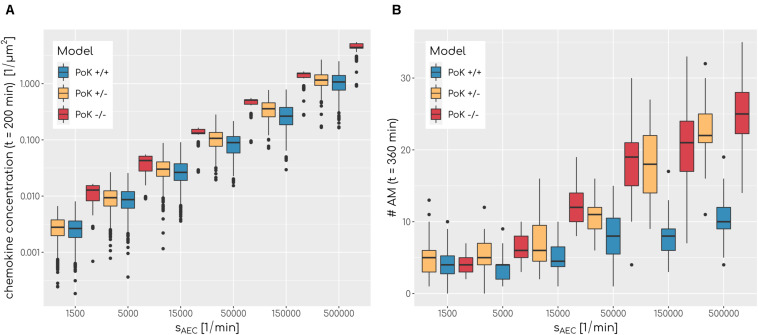
**(A)** Boxplot of mean chemokine concentration across the whole alveolar surface in the simulations at *t* = 200min for various secretion rates *s*_*AEC*_ and a fixed diffusion coefficient of *D* = 600μ*m*^2^min^−1^ for all three models. *N* = 1000. Each box represents to 25–75% quantile and central line represents the mean. **(B)** Boxplot of total sum of alveolar macrophages in the simulations at *t* = 360min for various secretion rates *s*_*AEC*_ and a fixed diffusion coefficient of *D* = 20μ*m*^2^min^−1^ for all three models. *N* = 1000. Each box represents to 25–75% quantile and central line represents the mean.

Our in-depth screening of the chemokine parameters for the lowest infection scores reveals that these are achieved for diffusion coefficient *D* = 60μ*m*^2^*min*^−1^ and secretion rate *s*_*AEC*_ = 50,000*min*^−1^ in the case of the PoK+/+ and the PoK+/− model whereas for the PoK−/− model the optimal chemokine parameters are shifted toward *D* = 20μ*m*^2^*min*^−1^ and *s*_*AEC*_ = 5000*min*^−1^. These findings reflect that in the PoK−/− model the chemokine dynamics is affected in two ways: First, in this model chemokine outflow is, in contrast to the PoK+/+ and PoK+/− models, prohibited and this leads to a higher accumulation of chemokines in the alveolus. Second, compared to the PoK+/+ and the PoK+/− models with PoK forming a sink for chemokines associated with a distortion of the chemokine gradient, in the PoK−/− model the chemokine gradient is more homogeneous and this may alter the migration patterns of AM into a migration behavior that is more directed. We analyzed the mean chemokine concentration across the whole alveolar surface after a simulation time of *t* = 200*min*. The difference in the mean chemokine concentration between the PoK+/+ model and the PoK−/− model yields on average a factor 4.4 for all secretion rates. This indicates that chemokines do indeed accumulate (see Figure 5A). It also explains the shift of the minimal infection score in the chemokine parameter space toward a lower secretion rate for the PoK−/− model compared to the other models, since the chemokine accumulation partly supersedes the secretion. Interestingly, also the PoK+/− model shows a slightly increased amount of chemokine in the alveolus compared to the PoK+/+ model, although the chemokine outflow of the models through PoK is identical. However, we find that this is caused by the chemokine uptake of AM with their distinguished spatial distribution in the PoK+/− and PoK+/+ models. Since in the PoK+/− model AM are initially distributed closer to the entrance ring (see Figure 2), where chemokine concentration is low, the chemokine uptake by AM is reduced compared to the PoK+/+ model (see Supplementary Figure S4).

**FIGURE 6:**
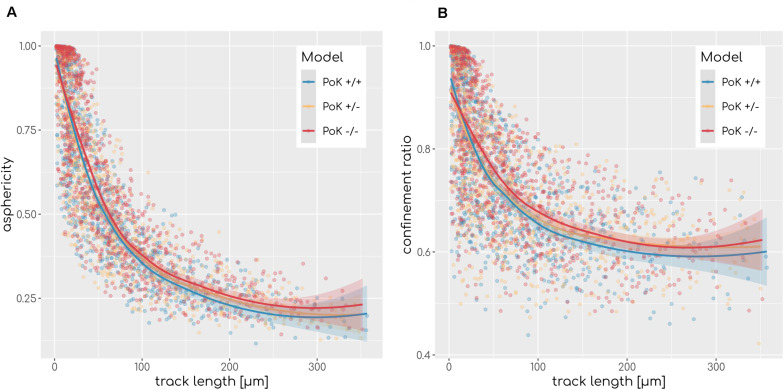
Asphericity **(A)** and confinement **(B)** ratio as a measurement of track straightness of each *AM*_*success*_ in relation to the track length for each of the three models for diffusion coefficient *D* = 20μ*m*^2^min^−1^ and secretion rate *s*_*AEC*_ = 1500min^−1^. Regression line represents LOESS fit and the shaded area represents the estimations standard error.

Next, we investigated the question if changes in the chemokine profile affect the migration behavior of AM in a measurable way. To this end, we analyzed the cell tracks of all successful *AM*_*success*_ using established cell track analysis methods by computing the asphericity ratio *A* and the confinement ratio *C* of cell tracks (Mokhtari et al., 2013). The asphericity ratio is computed by the ratio of the longest and shortest axes of the enclosing ellipse for a track of *N* time points. The confinement ratio $C=\frac{1}{N^{2}}\,\sum_{m,n}\frac{l\,\left(m,n\right)}{d\,\left(m,n\right)}$ for a track of time-ordered points *t*_1_…*t*_*N*_ is computed by the mean ratio of track length *l*(*m*,*n*) = $\sum_{i=m}^{n-1}d\,\left(i,i+1\right)$ to the Euclidian distance *d*(*m*,*n*) of track points *m* and *n* with *m*,*n* ∈ [1,*N*]. These two measures quantify the straightness of a track on a continuous scale with *A*,*C* ∈ [0,1], where values of 1 are indicative for perfectly straight tracks, while spatially confined tracks are scored with values close to 0. Since these measures are affected by the track length, we calculated the *A* and *C* for each track as a function of the track length (see Figure 6). To test for differences we applied a local regression (LOESS) analysis, which revealed that all three models show highly similar asphericity and confinement ratios with each fit being within the others fits’ standard error (see Figure 6). Thus, this analysis predicts no differences in the migration patterns of successful AM for the three PoK models.

### Accumulation of AM Limited in Presence of PoK Passageways

The chemokine-mediated recruitment of AM is naturally associated with their accumulation in the alveolus, because it is unlikely that AM following the chemokine gradient will leave the alveolus before the infection is cleared. Our simulations allow for a comparative analysis of the time-dependent increase in the AM number for the three PoK models. This analysis confirmed that AM accumulate in a way that depends on the exact chemokine parameters. In Figure 5B it can be seen that a higher secretion rate *s*_*AEC*_ is associated with higher accumulation of AM. For e.g., optimal chemokine parameters (*D* = 60μ*m*^2^*min*^−1^, *s*_*AEC*_ = 50,000*min*^−1^) the mean number of AM in the PoK+/+ model increases up to 177% compared to random walk migration of AM; this increase is even higher with up to 254% and 413%, respectively, for the PoK+/− and PoK−/− model. This accumulation is due to AM recruitment by the chemokine gradient, which depends on the model scenarios. Since AM are attracted toward the conidium, they tend to stay in the alveolus instead of leaving the alveolus by migrating against the gradient. Interestingly within the range of AM numbers observed in our simulations, accumulation of AM is only weakly correlated with lowering the infection score, i.e., we found a Pearson correlation coefficient of −0.41 (see Supplementary Figure S5A). A massive recruitment of AM toward a single alveolus within the pulmonary tissue might increase the risk for unresolved infections in other alveoli keeping in mind that the lung is constantly exposed to various pathogens simultaneously. Therefore, high levels of AM accumulation in a specific alveolus should be generally avoided to keep the immune system in a state of flexible responsiveness.

**FIGURE 7:**
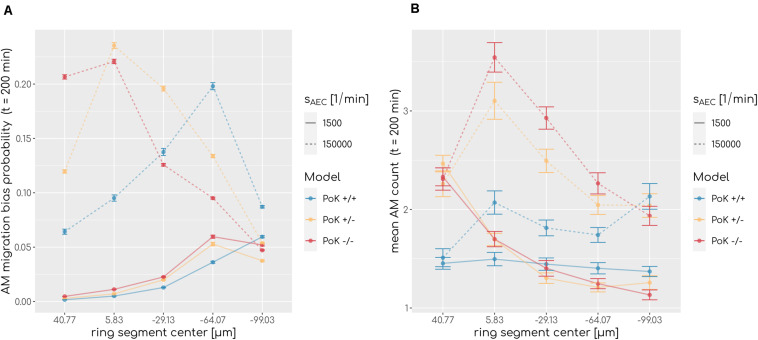
**(A)** Probability of an alveolar macrophage to migrate biased toward the chemokine gradient and **(B)** accumulation of alveolar macrophages along the main axis of the alveolus from entrance ring (59 μm) to pole (-116.5 μm) for the PoK+/+ model and the PoK−/− model for selected secretion rates and diffusion coefficient *D* = 20μ*m*^2^*min*^−1^. Each data point refers to an average over ring segments with equal area of 25,583 μm^2^.

To understand why AM accumulation occurs, it is important to put the chemokine secretion induced at the conidium site into perspective. Since the evolving chemokine profile is strongly influenced by the random position of the conidium in the alveolus, we decided to make the three different models comparable by locating the conidium at a fixed position in each simulation. This position was chosen to be at the pole of the alveolus, i.e., at the symmetry point of the alveolus with largest distance to the entrance ring (see Figure 1). The induced chemokine profile attains highest concentration values at the pole and lowest values at the alveolar entrance ring; thus, the concentration gradient has its steepest slope in between depending on the applied chemokine parameters, i.e., the diffusion coefficient and the secretion rate. While at the point of steepest slope AM follow the chemokine gradient with highest probability, they tend to switch to predominantly random walk migration close to the pole and the entrance ring (see Figure 7A). We observed in our simulations that the higher the chemokine secretion rate, the closer is the point of steepest slope shifted to the entrance ring. As a result, AM entering the alveolus quickly migrate to this region and will most likely remain there. As a consequence, AM are less likely to leave the alveolus or detect the conidium at the pole position (see Figure 7B). This effect due to the chemokine accumulation is observed in all three models, but is most pronounced in the PoK−/− model and explains the increased AM numbers (see Supplementary Figure S6). While increased AM numbers may be thought to be associated with a lower infection score, the quantitative evaluation of our computer simulations revealed this correlation to be only weak, independent whether the conidium was positioned randomly or at the pole (see Supplementary Figures S5, S7). For a more detailed comparison between the random conidium positioning model and the setup with a conidium fixed at the alveolus pole we refer to the Supplementary Material Section 1.3.

## Discussion

In the present study, we investigated the impact of Pores of Kohn (PoK) on the clearance of *Aspergillus fumigatus* infections in the human lung. To this end, we performed computer simulations based on an established hybrid agent-based virtual infection model (Pollmächer and Figge, 2014, 2015; Blickensdorf et al., 2019). This framework represents a realistic, spatio-temporal three-dimensional model that was carefully designed based on available experimental data from literature on the human alveolus. It incorporates alveolar macrophages (AM) and chemokine signaling released by AEC to allow screening for physiologically relevant signaling parameters as well as to quantitatively investigate infection clearance. We developed three model setups to investigate how infection clearance would be affected by passaging of AM or chemokines through PoK: the PoK+/+ model, which allows for chemokine and AM exchange, the PoK+/− model, which does allow only for chemokine exchange, and the PoK−/− model, which does not allow for chemokine and AM exchange.

We could show that, over the scanned range of chemokine parameters significant differences in the infection clearance between the models exist. However, the impact of PoK on the spatio-temporal infection dynamics has only a small effect size: The absolute differences in the infection scores that were computed from our simulations are smaller than 0.05 for 72% of all comparisons. This suggests that infection clearance can be comparably well achieved within the PoK+/+ model as well as the PoK−/− and PoK+/− models. We conclude that AM recruitment through PoK plays only a minor role with regard to infection clearance. However, we found that the PoK+/+ model suggests that infection clearance is independent of the position of the conidium, whereas in the PoK+/− and the PoK−/− model conidia detection was more efficiently realized close to the alveolar entrance ring than at the alveolus pole (see Figure 4). Thus, a conidium at the pole would impose a higher infection risk for the host if AM could not migrate through PoK. Although the assumption of a uniform random distribution of the conidium position after inhalation is reasonable, a spatial distribution of conidium positions that is biased in some way may affect the infection score in favor of one model. For example, Xi and Talaat (2019) suggest that conidia reside in close proximity to the alveolar entrance ring, where the PoK+/− and PoK−/− models have a detection advantage, since all AM enter through the alveolar entrance ring leading to a higher density of AM in this region (see Figure 4). Our simulations also showed that, without pathogens present in the alveolus, AM migration trough PoK allowed for a uniform distribution of AM on the alveolar surface, whereas prohibited AM migration through PoK leads to an accumulation of AM in the proximity of the entrance ring.

Besides differences in the infection score between the three models, our simulations revealed that other dynamics are affected by different properties of PoK. In the PoK−/− model the prohibited outflow of chemokine through PoK causes an accumulation of chemokines. As a consequence, the lowest infection score is achieved at a different chemokine parameter regime with a lower secretion rate *s*_*AEC*_ compared to the PoK+/+ and PoK+/− models. Since the true secretion rate still needs to be determined experimentally, it could indeed be possible that the PoK−/− model outperforms the other two models in terms of infection clearance. Furthermore, our simulations demonstrate the effect of AM accumulation caused by chemokine signaling and show its dependency on the properties of PoK. Although this effect is dependent on the secretion rate *s*_*AEC*_ and the diffusion coefficient *D* and can be observed in all three models, relatively high AM accumulation was present in the PoK+/− and even stronger in the PoK−/− model. This AM accumulation effect imposes a higher risk for undetected pathogens. AM that are recruited to a site of infection may be effectively missing in other alveoli of the lung. The lung is an organ, which is constantly confronted with various pathogens simultaneously, implying that the AM availability is a crucial factor for a fast response of the immune defense. An unnecessarily excessive recruitment of AM, therefore, may be disadvantageous for the host and PoK that allow for AM passage can play an important role in this regulation.

One of the most recent and detailed studies on the function of PoK in the human lung discusses the behavior of alveoli during inflation (Namati et al., 2008). Namati et al. (2008) further developed a hypothesis according to which alveoli are changing their shape in reaction to air pressure changes and combined it with the hypothesis that alveoli are consecutively inflated and thus regulate the air pressure. A key role in this mechanism would be played by PoK, which might open and close due to these pressure changes. As a result, AM would have to squeeze though closing and opening PoK. The latter processes would happen fast according to the typical human breathing frequency of 12−18*min*^−1^ (Barrett et al., 2012). A typical AM speed of 4 μ*mmin*^−1^ suggests that for migrating through a PoK of a few micrometers in length, AM would need in the order of 1 min for PoK passage (Glasgow et al., 1989). Thus, AM passage through PoK would be interrupted by frequent closing and opening of PoK. One option to combine both hypotheses of AM passaging through PoK and frequent PoK opening would be that AM migration is air-flow assisted due to the associated high changes in the air pressure in PoK, as suggested by Namati et al. (2008) A similar argumentation can be found by Oldham and Moss (1989) arguing that the opening of a PoK due to inhalation may result in bursting of the surfactant layer. An AM residing at such a spot may thus be accelerated and “pushed” through a PoK. While Peão et al. (1993) as well as Bastacky and Goerke (1992) present images of AM within PoK, the quantitative analysis of the infection clearance in the three different virtual infection scenarios allows for the statement that AM passaging is not necessary for low infection scores but imposes the positive effect of homogeneous AM distributions in the alveolus.

In the future, our understanding of the role of PoK may be deepened by systems biology approaches that take into account aspects of the mechanics of alveolar ventilation as well as other immune cell types involved in the host-pathogen interaction between humans and *A. fumigatus*. In particular, Bozza et al. (2002) observed that dendritic cells (DCs) are as well involved in the immune response against *A. fumigatus* during the first hours post-infection: DCs were shown to (i) internalize conidia and hyphae (ii) discriminate between the different forms regarding cytokine production, (iii) undergo functional maturation upon migration to draining lymph nodes and spleens, and (iv) instruct local and peripheral T-helper cell reactivity against *A. fumigatus*. Furthermore, it is known that polymorphonuclear neutrophils, which may be recruited by AM from the blood stream (Pollmächer et al., 2016), are involved in the immune response during pulmonary aspergillosis and provide an arsenal of immune effector mechanisms against *A. fumigatus* (Brakhage et al., 2010). In this context, it will be important to investigate the system dynamics as a function of the number of immune cells that are estimated to be resident in or recruited to the alveolus. Additionally it has been demonstrated that AEC are able to ingest and phagocytose conidia (Osherov, 2012). Furthermore, the hybrid agent-based framework can be applied to also simulate hyphal growth of the fungus at later time points and invasion into the blood vessels. However, modeling such complex mechanisms requires a firm experimental data basis, which might be provided by imaging experiments that can nowadays be realized using lung-on-chip models (Mosig, 2017; Deinhardt-Emmer et al., 2020; Schicke et al., 2020).

## Data Availability Statement

The raw data supporting the conclusions of this article will be made available by the authors, without undue reservation, to any qualified researcher.

## Author Contributions

MF conceived and designed this study and provided computational resources. MB did the data processing, implementation and application of the computational algorithm. MB, ST, and MF evaluated and analyzed the results of this study, drafted the manuscript, revised it critically for important intellectual content and final approval of the version to be published, and agreed to be accountable for all aspects of the work in ensuring that questions related to the accuracy or integrity of any part of the work are appropriately investigated and resolved. All authors contributed to the article and approved the submitted version.

## Conflict of Interest

The authors declare that the research was conducted in the absence of any commercial or financial relationships that could be construed as a potential conflict of interest.
