# Supplementary material for: Hybrid Agent-Based Modeling of Aspergillus fumigatus Infection to Quantitatively Investigate the Role of Pores of Kohn in Human Alveoli
Source: Front Microbiol. 2020 Aug 12;11:1951. doi: 10.3389/fmicb.2020.01951 (PMC7438790; doi:10.3389/fmicb.2020.01951)
Supplement: Supplementary file 4 [file Data_Sheet_1.pdf]

Supplementary Material

Hybrid agent-based modeling of *Aspergillus fumigatus* infection to quantitatively investigate the role of Pores of Kohn in human alveoli

Marco Blickensdorf, Sandra Timme and Marc Thilo Figge *

***Correspondence:** Corresponding Author thilo.figge@leibniz-hki.de

1 Supplementary Methods

1.1 Model Comparison by Log-Rank Test

To evaluate differences of the three models we compared the first passage time (FPT) *i.e.* the time needed for each simulation to detect the conidium by an AM. Therefore, we performed 1000 simulations for each set of parameters yielding a FPT distribution. Since this corresponds to a survival curve of the conidium, we applied a three-grouped log-rank test (Mantel-Haenszel) as typically used for survival analysis (4). A significance level of 0.05 was applied.

1.2 Diffusion Coefficient Scanning Range

The hybrid agent-based model comprises chemokine signaling released by AEC where the conidium is located. Since physiological properties of this chemokine are unknown, it is necessary to scan chemokine properties, such as the diffusion constant and the secretion rate by AEC, within reasonable ranges. The diffusion coefficient can be determined by the Stokes-Einstein equation if the radius of the molecule and the viscosity of medium is known. The mass of known chemokines, such as MCP-1 and MIP, ranges from $8-12$ kDa with an approximated molecular radius of $1.3-1.5\,nm$ (5). In contrast, the viscosity value of the lung surfactant is harder to delimit. King et al. measured the viscosity of calf lung surfactant and give a range of $3-30\,cP$ depending on the, also unknown, shear rate and molecule concentration within the surfactant (6). Other sources yield values from $21-70000\,cP$ for pulmonary mucus of patients with lung diseases like cystic fibrosis or infections like bronchitis (7). Given this huge viscosity range, the Stokes-Einstein equation yields diffusion coefficients from $0.13\,\mu m^2 min^{-1}$ for high viscosity as in mucus up to $3500\,\mu m^2 min^{-1}$ in calf surfactant. Thus, since we consider lung conditions without any pre-existing infections or diseases in our study, we chose to screen the diffusion coefficient D from $20\,\mu m^2 min^{-1}$ up to

$6000\ \mu m^2 min^{-1}$. This range coincides with our earlier estimates for chemokine diffusion in water (2).

1.3 Conidia Positioning at Alveolus Pole Favors PoK+/+ Model

For a better comparability of the three models we also performed simulations, in which the conidia was not placed uniform randomly in the alveolus but at the pole of the alveolus *i.e.* at the symmetry point of the alveolus with largest distance to the entrance ring (see Fig. 1). Although this model setup represents an extreme case, which puts a disadvantage on the PoK+/- and PoK-/- model it allows for a comparison to the model with random conidia positioning. This analysis revealed that the minimal IS dropped in each of the three models but remained close to the model with random conidia positioning of $IS \sim 0.1$ only for the PoK+/+ model (see Supplementary Fig. S7). This is in accordance with our findings from the analysis of IS depending on the position of the conidium (see Fig. 4) and can be explained by the altered migration distance between the conidium and the alveolus boundaries. Whereas in the random positioning model approximately 60% of the conidia are found by AM, which were not recruited into the alveolus at PoK or entrance ring but were present from the start of the simulation. The simulations with a conidium fixed at the pole yielded fractions of 0.75 ± 0.04 in the PoK+/+ model and 0.95 ± 0.06 (0.94 ± 0.05) in the PoK+/- (PoK-/-) model indicating a smaller contribution of AM recruitment. An analysis of the accumulation of chemokines and AM shows that these effects occur also in the simulations where conidia were placed at the alveolus pole (see Supplementary Fig. S6).

2 Supplementary Figures

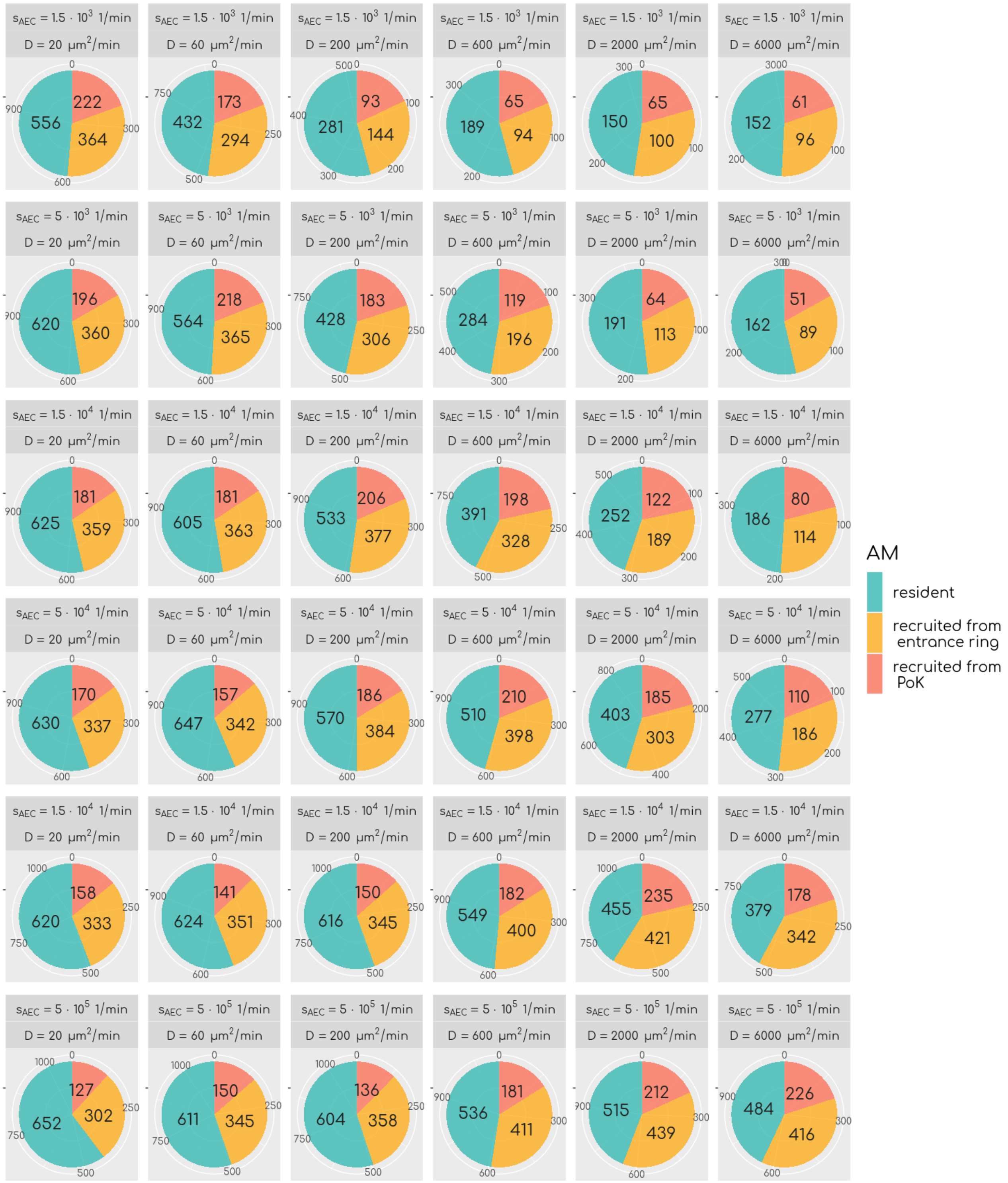

Supplement Figure 1: Origin of AM, which were successful in detecting a conidium for each model and all scanned chemokine parameters for the PoK+/+ model.

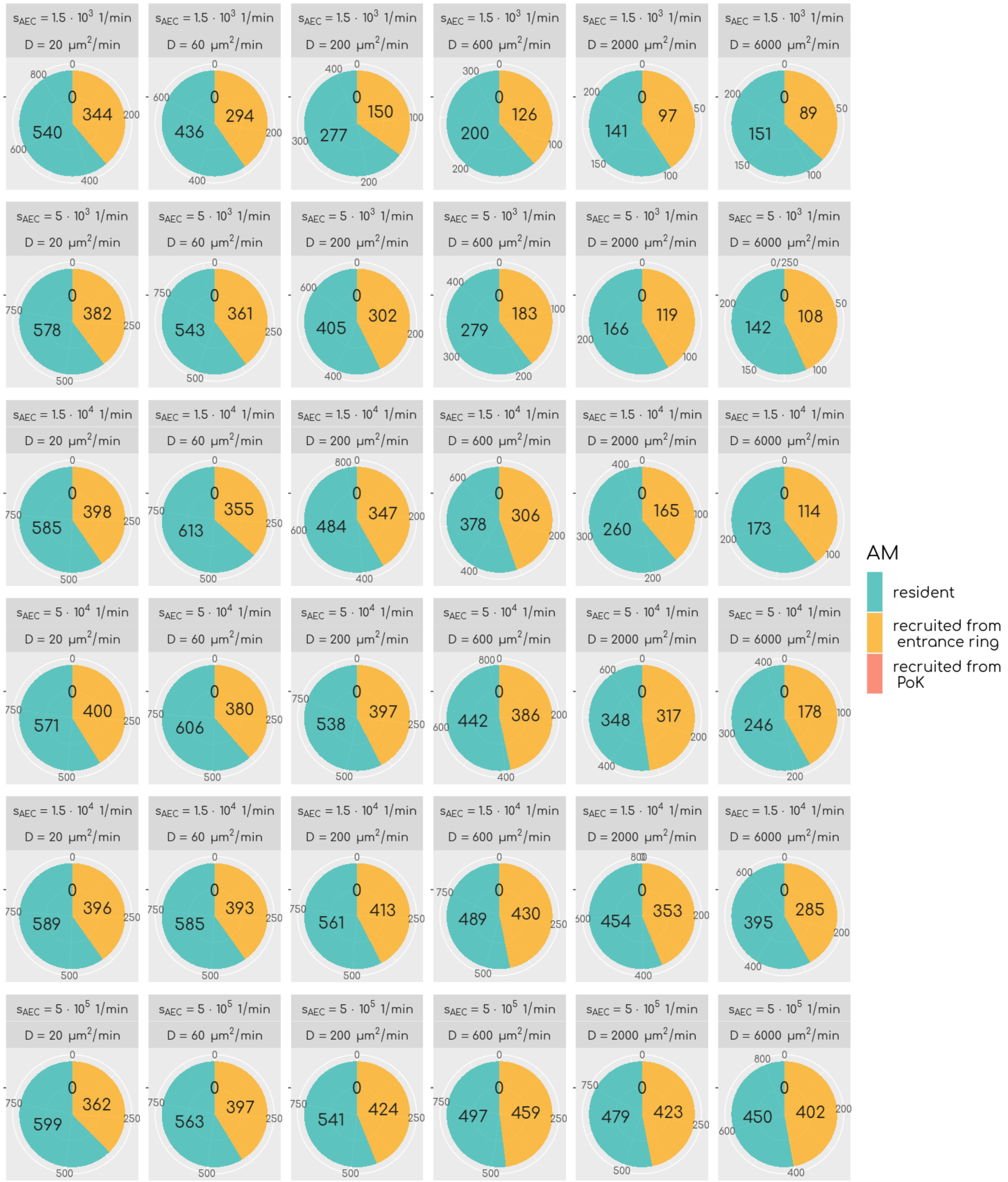

Supplement Figure 2: Origin of AM, which were successful in detecting a conidium for each model and all scanned chemokine parameters for the PoK+/- model.

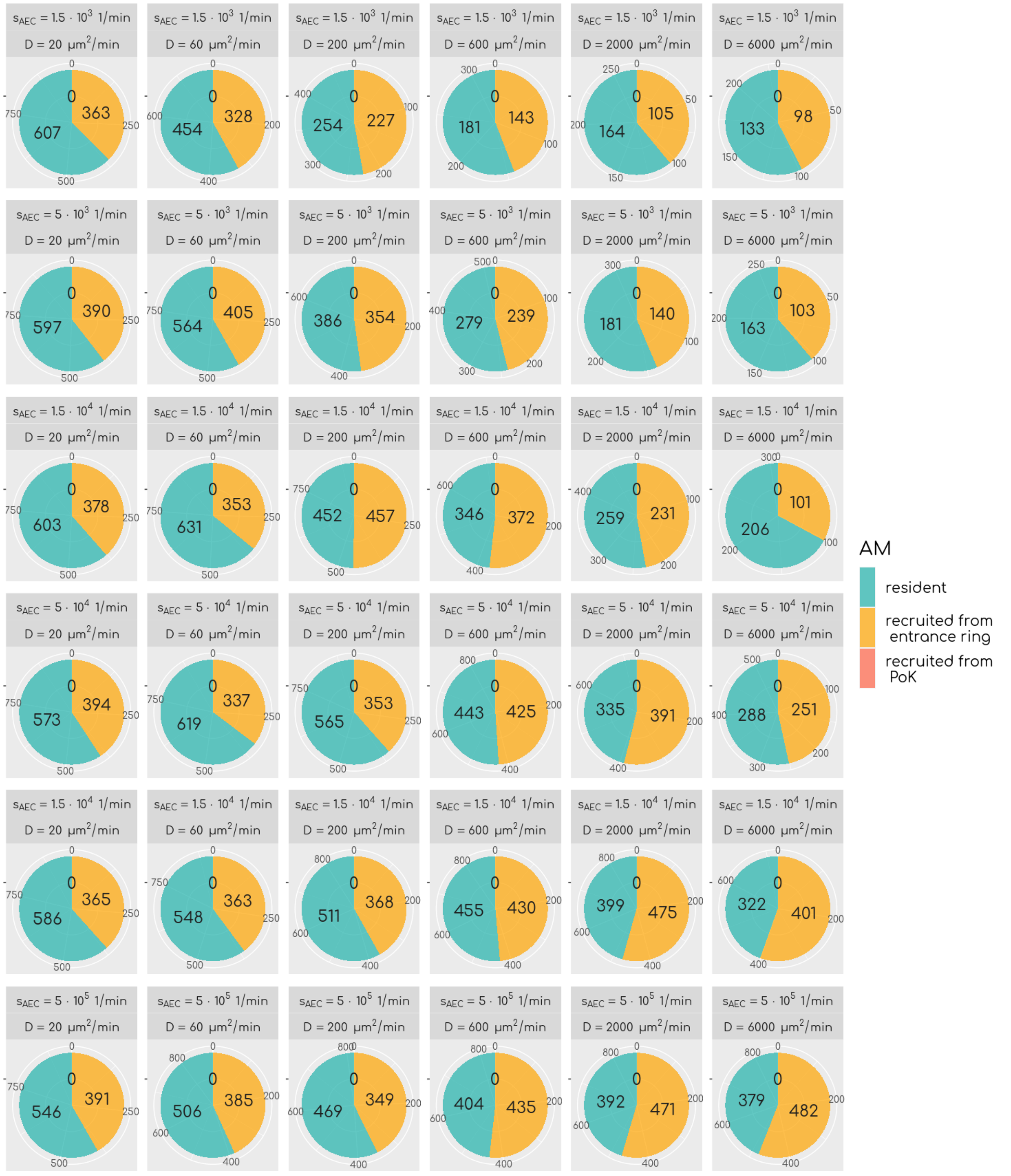

Supplement Figure 3: Origin of AM, which were successful in detecting a conidium for each model and all scanned chemokine parameters for the PoK-/- model.

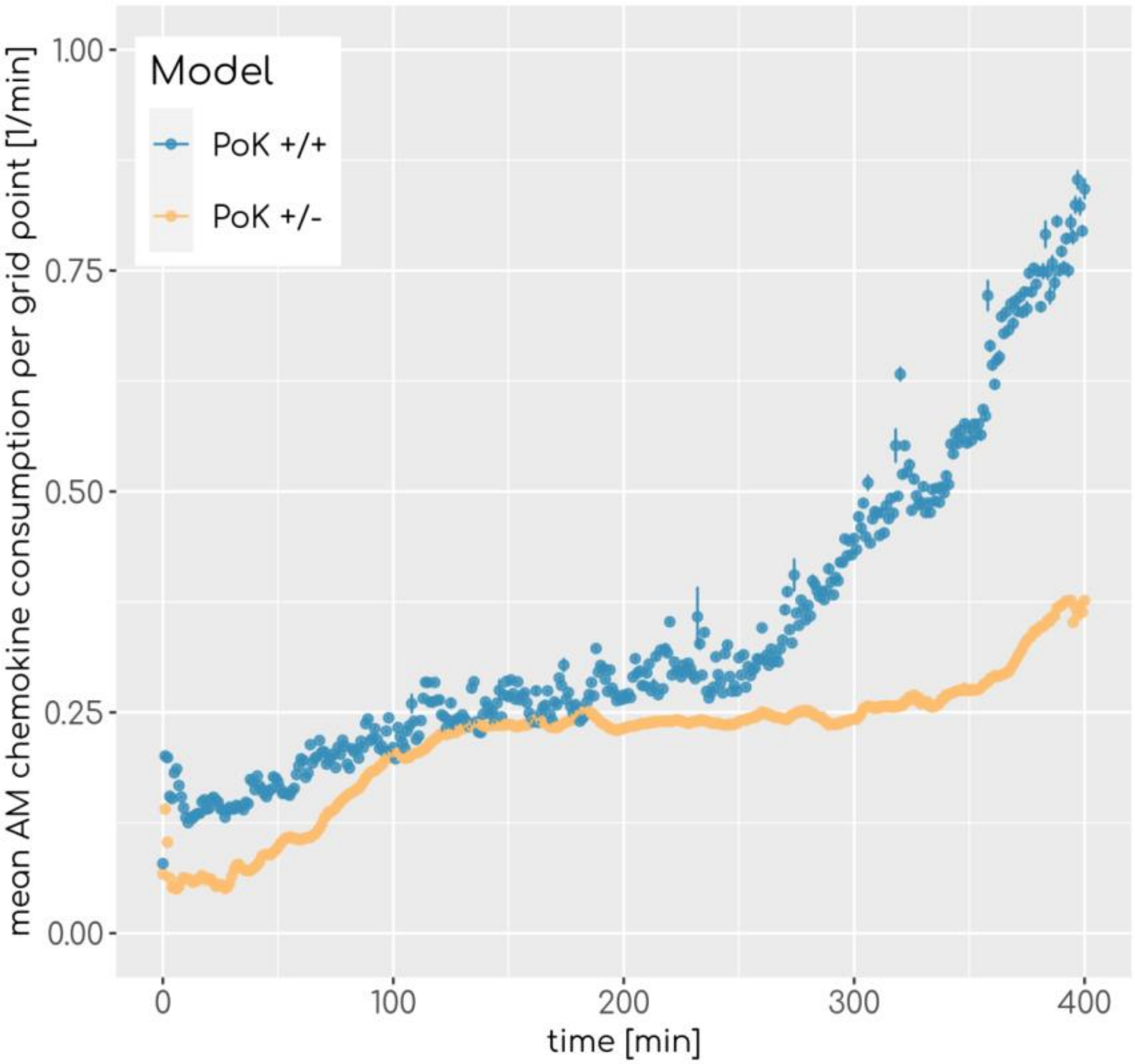

Supplement Figure 4: Mean consumption of chemokines per grid point per minute by AM. $\boldsymbol{s_{AEC}} = \mathbf{1500}\ \boldsymbol{min^{-1}}, \boldsymbol{D} = \mathbf{20}$ μ$\boldsymbol{m^2 min^{-1}}$. Vertical bars represent the standard error.

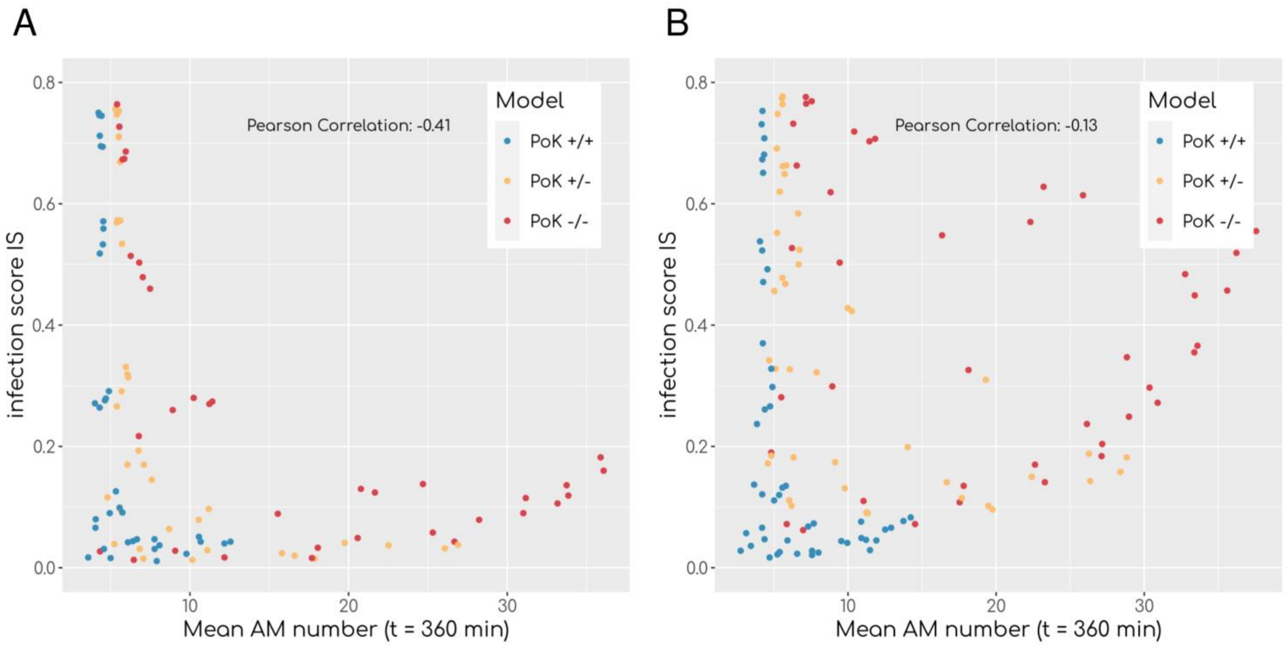

Supplement Figure 5: Correlation of AM number of the simulation at time $t = 360\,min$ to the infection score for A) random conidia positioning and B) fixed conidia positioning at pole of alveolus. The Pearson correlation coefficient was calculated as -0.41 and -0.13.

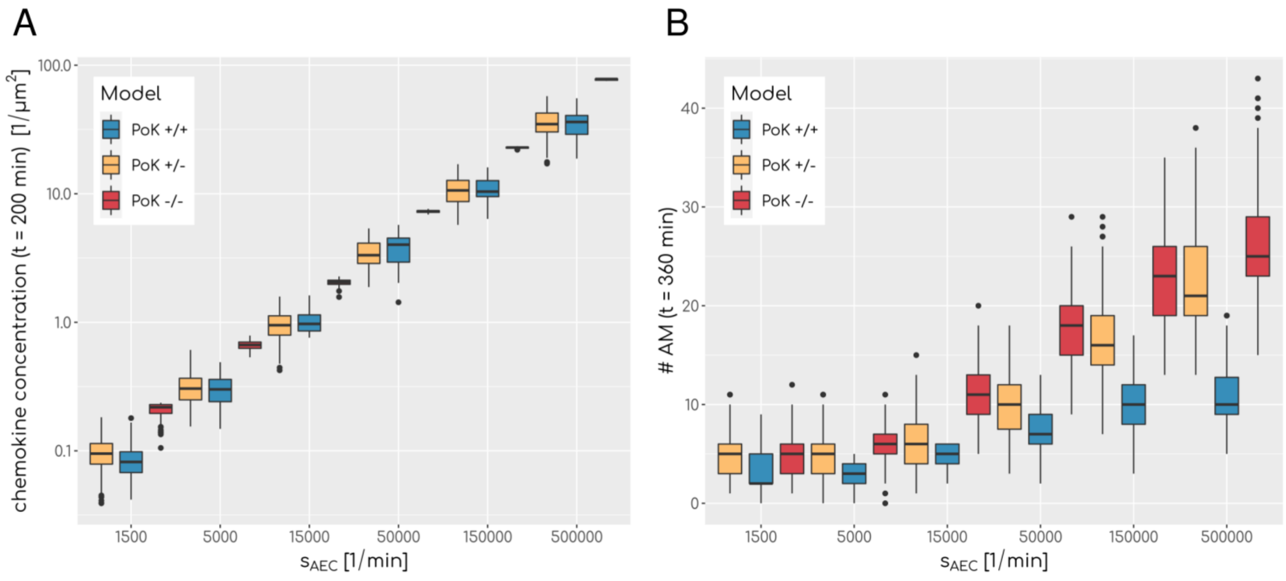

Supplement Figure 6: Results for simulations with fixed conidium placing at pole of alveolus. A) Boxplot of the mean chemokine concentration across the whole alveolar surface in the simulations at $t = 200\,min$ for various secretion rates s_{AEC} and a fixed diffusion coefficient of $D = 600\ \mu m^2 min^{-1}$ for all three models. N = 1000. Each box represents to 25%-75% quantile and central line represents the mean. B) Boxplot of total sum of alveolar macrophages in the simulations at $t = 360\,min$ for various secretion rates s_{AEC} and a fixed diffusion coefficient of $D = 20\ \mu m^2 min^{-1}$ for all three models. N = 1000. Each box represents to 25%-75% quantile and central line represents the mean.

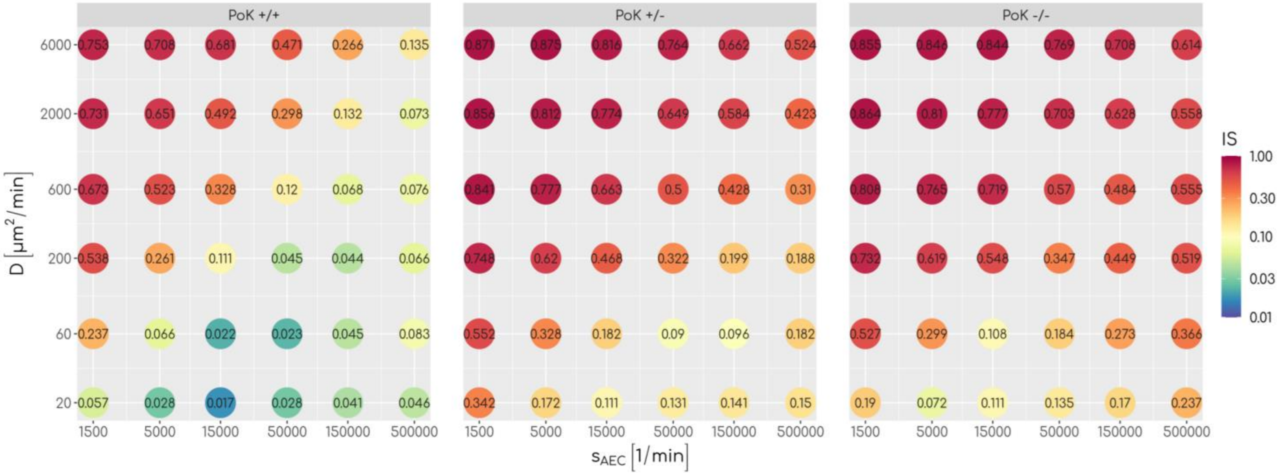

Supplement Figure 7: The infection score represented in a color-coded fashion as a function of all scanned combinations of chemokine parameters for simulations with a conidium placed at the alveolus pole. Numbers represent the respective infection scores.

3 Supplementary Videos

Each video shows a simulation with a randomly placed conidium of the respective three models. Simulations end when an AM detects a conidium.

3.1 Supplementary Video 1 – Visualization of PoK+/+ Model

Visualization of a to-scale human alveolus in the hybrid agent-based model. The alveolar entrance ring (left) and Pores of Kohn (black) represent entry/exit points for alveolar macrophages (green) and chemokine flow (white isolines) induced by the alveolar epithelial cell where the conidium (red) is located. Alveolar surface is covered with epithelial cells of type 1 (yellow) and type 2 (blue).

3.2 Supplementary Video 2 – Visualization of PoK+/- Model

Visualization of a to-scale human alveolus in the hybrid agent-based model. The alveolar entrance ring (left) and Pores of Kohn (black) represent entry/exit points for alveolar macrophages (green) and chemokine flow (white isolines) induced by the alveolar epithelial cell where the conidium (red) is located. Alveolar surface is covered with epithelial cells of type 1 (yellow) and type 2 (blue).

3.3 Supplementary Video 3 – Visualization of PoK-/- Model

Visualization of a to-scale human alveolus in the hybrid agent-based model. The alveolar entrance ring (left) and Pores of Kohn (black) represent entry/exit points for alveolar macrophages (green) and chemokine flow (white isolines) induced by the alveolar epithelial cell where the conidium (red) is located. Alveolar surface is covered with epithelial cells of type 1 (yellow) and type 2 (blue).

4 Supplementary References

1. Pollmächer J, Figge MT. Agent-based model of human alveoli predicts chemotactic signaling by epithelial cells during early Aspergillus fumigatus infection. PLoS One. 2014;9(10):e111630.

2. Pollmächer J, Figge MT. Deciphering chemokine properties by a hybrid agent-based model of Aspergillus fumigatus infection in human alveoli. Front Microbiol. 2015;6:503.

3. Blickensdorf M, Timme S, Figge MT. Comparative assessment of aspergillosis by virtual infection modeling in murine and human lung. Front Immunol. 2019 Feb 5;10:142.

4. Harrington DP, Fleming TR. A class of rank test procedures for censored survival data. Biometrika. 1982; 69:553-66

5. Erickson HP. Size and shape of protein molecules at the nanometer level determined by sedimentation, gel filtration, and electron microscopy. Biological Procedures Online. 2009 May 15;11:32-51

6. King DM, Wang Z, Kendig JW, Palmer HJ, Holm BA, Notter RH. Concentration-dependent, temperature-dependent non-Newtonian viscosity of lung surfactant dispersions. Chem Phys Lipids. 2001 Jul;112(1):11-9.

7. Chen Z, Zhong M, Luo Y, Deng L, Hu Z, Song Y. Determination of rheology and surface tension of airway surface liquid: A review of clinical relevance and measurement techniques. Respiratory Research. 2019: 20, 274.
